# Arginase 1 is a marker of protection against illness in contacts of leprosy patients

**DOI:** 10.1038/s41598-022-11944-9

**Published:** 2022-05-12

**Authors:** Rhana Berto da Silva Prata, Mayara Abud Mendes, Vinicius Cardoso Soares, Jaqueline França-Costa, Anna Maria Sales, Nádia Cristina Duppré, Valéria de Matos Borges, Tatiana Pereira da Silva, Patricia Torres Bozza, Marcelo Torres Bozza, Euzenir Nunes Sarno, Milton Ozório Moraes, Gilberto Marcelo Sperandio da Silva, Roberta Olmo Pinheiro

**Affiliations:** 1 Leprosy Laboratory, Oswaldo Cruz Institute, Oswaldo Cruz Foundation, Avenida Brasil, 4365, Manguinhos, Rio de Janeiro, RJ 21040-360 Brazil; 2 Inflammation and Immunity Laboratory, Federal University of Rio de Janeiro, Rio de Janeiro, RJ Brazil; 3 Immunopharmacology Laboratory, Oswaldo Cruz Institute, Oswaldo Cruz Foundation, Rio de Janeiro, RJ Brazil; 4 Immunology Service, Professor Edgar Santos University Hospital, Federal University of Bahia, Salvador, Brazil; 5 Inflammation and Biomarkers Laboratory, Gonçalo Moniz Institute, Oswaldo Cruz Foundation, Salvador, BA Brazil; 6 Clinical Research Laboratory in Chagas Disease, National Institute of Infectology Evandro Chagas, Oswaldo Cruz Foundation, Rio de Janeiro, RJ Brazil

**Keywords:** Immunology, Infectious diseases

## Abstract

Leprosy household contacts are generally more prone to develop the disease compared to the general population. Previous studies have demonstrated that genes related to the alternative activation (M2) profile in macrophages are associated with the increased bacillary load in multibacillary leprosy patients (MB), and that contacts of MB patients have a higher risk of contracting the disease. In addition, positive serological responses to PGL-1 or LID-1 are associated with a higher risk of disease. We performed a 5-year follow-up of contacts of leprosy patients and evaluated the pattern of gene and protein expression in cells from contacts that developed leprosy during this period. Leprosy household contacts had decreased soluble CD163 and heme oxygenase 1 (HO-1) serum levels when compared with healthy donors and leprosy patients. In contrast, arginase 1 activities were higher in contacts when compared with both healthy donors and leprosy patients. Of the contacts, 33 developed leprosy during the follow-up. Gene expression analysis revealed reduced *ARG1* expression in these contacts when compared with contacts that did not develop disease. Arginase activity was a good predictive marker of protection in contacts (sensitivity: 90.0%, specificity: 96.77%) and the association with serology for anti-PGL-1 and anti-LID-1 increased the sensitivity to 100%. Altogether, the data presented here demonstrate a positive role of arginase against leprosy and suggest that the evaluation of arginase activity should be incorporated into leprosy control programs in order to aid in the decision of which contacts should receive chemoprophylaxis.

## Introduction

The implementation of the World Health Organization (WHO) multidrug therapy in the 1980s contributed to a decrease in the global burden of people affected by leprosy. However, in some countries like India and Brazil, the new case detection rate remained static, probably due to transmission of *Mycobacterium leprae* from existing untreated cases and active transmission in the community^1^.

According to the WHO, contacts of patients with leprosy are individuals who have close or intimate association with the patients. Contacts that share the same residence are the most easily identified, and these have a risk of contracting the disease almost four times greater than a non-contact^2,3^. Since household contacts of leprosy patients are at highest risk, there is a recommendation for the use of chemoprophylaxis as preventive treatment for these individuals. Although effective, more chemoprophylaxis research is needed to identify enhanced medication regimens and determine specific approaches per contact type, as described by Schoenmakers and colleagues^4^. Compulsory treatment for all contacts may become too expensive for the health system though, so the search for biomarkers that can predict a subclinical infection could contribute to a cost-effective chemoprophylactic strategy.

Previous studies have shown some high-risk factors associated with leprosy development in contacts, such as the clinical form of the index case^5^, serum-positivity for phenolic glycolipid 1 (PGL-1)^6^, PCR positivity for *M. leprae* DNA^7,8^, and non-BCG vaccination^9–11^. However, none of these have yet been proven to be a good parameter for the identification of new cases, so the study of other potential biomarkers for the disease is indispensable for this identification and possible early clinical diagnosis and drug intervention.

To understand the mechanisms that are pivotal for the development of leprosy in *M. leprae-*infected individuals, longitudinal follow-up of household contacts is a vital source of information. This longitudinal follow-up enables the identification of household contacts developing disease from those that are exposed to *M. leprae* but do not develop disease. Van Hooij and Geluk^12^ hypothesized that in contacts there is a constant battle between the host and the bacterium, and in household contacts that do not develop disease, the balance is in favor of the host, whereas in those who develop disease, the pathogen succeeds in establishing the infection.

Our previous studies have demonstrated that higher bacillary loads in leprosy patients are positively correlated with the presence of macrophages with an anti-inflammatory phenotype, associated with the expression of the scavenger receptor CD163, the enzyme heme oxygenase 1 (HO-1), and arginase 1^13–15^. Here, we evaluated if anti-inflammatory molecules associated with more susceptible macrophages could be used as biomarkers to identify the contacts who will develop disease, which could be useful not only in the detection of leprosy in preclinical stages of the disease but also to identify the targets for chemoprophylactic strategies.

## Results

### Arginase activity is increased in serum samples from leprosy contacts

**Figure 1:**
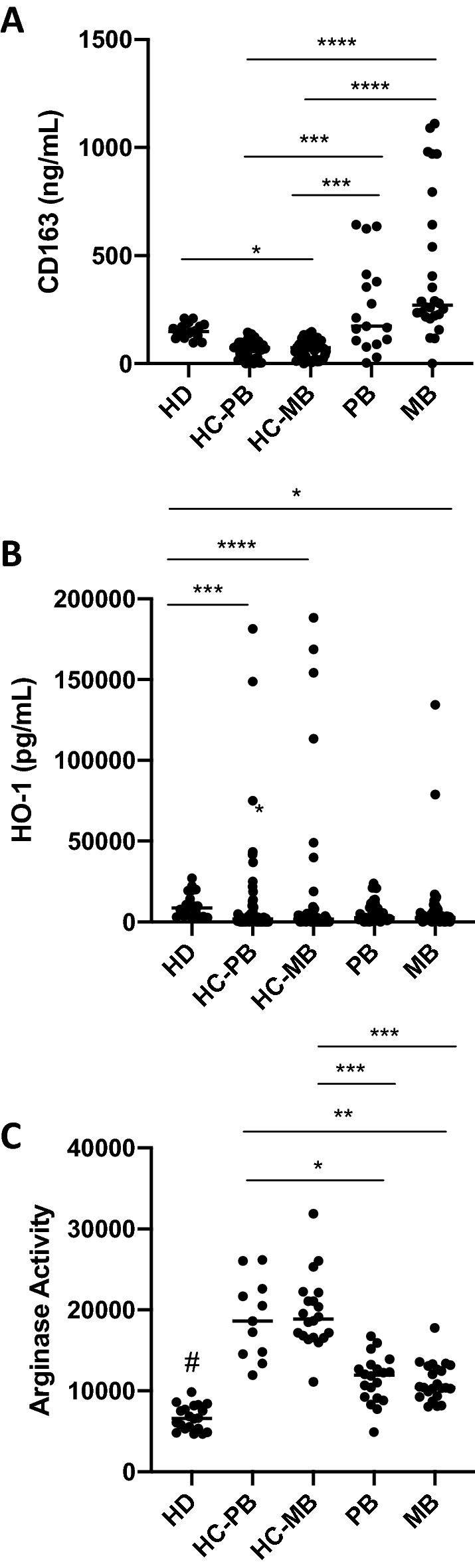
Contacts of leprosy patients present higher arginase activity than healthy controls and patients. Serum from healthy donors of endemic areas (HD, n = 18), paucibacillary patients (PB, n = 42), multibacillary patients (MB, n = 59), household contacts of PB patients (HC-PB, n = 58), and household contacts of MB patients (HC-MB, n = 83) were collected and levels of soluble (**A**) CD163 and (**B**) HO-1 were evaluated by ELISA. In addition, (**C**) arginase activity was evaluated. *p ≤ 0.05, **p ≤ 0.01, ***p ≤ 0.001, ****p ≤ 0.0001.

Levels of sCD163 and HO-1 were determined by ELISA in sera from healthy donors (HD, n = 18), paucibacillary patients (PB, n = 42), multibacillary patients (MB, n = 59), household contacts of PB patients (HC-PB, n = 58), and household contacts of MB patients (HC-MB, n = 83). In addition, arginase activity was evaluated. As observed in Fig. 1, sCD163 serum levels were significantly increased in leprosy patients when compared with the HC-PB and HC-MB groups. HO-1 levels were reduced in both groups of contacts when compared with HD. In addition, HD presented higher levels of HO-1 when compared with MB patients. Arginase activity was increased in both HC-PB and HC-MB groups when compared with the leprosy patient groups. These data indicate that arginase activity may be useful to discriminate contacts and patients.

### Arginase is a marker of protection against leprosy

**Figure 2:**
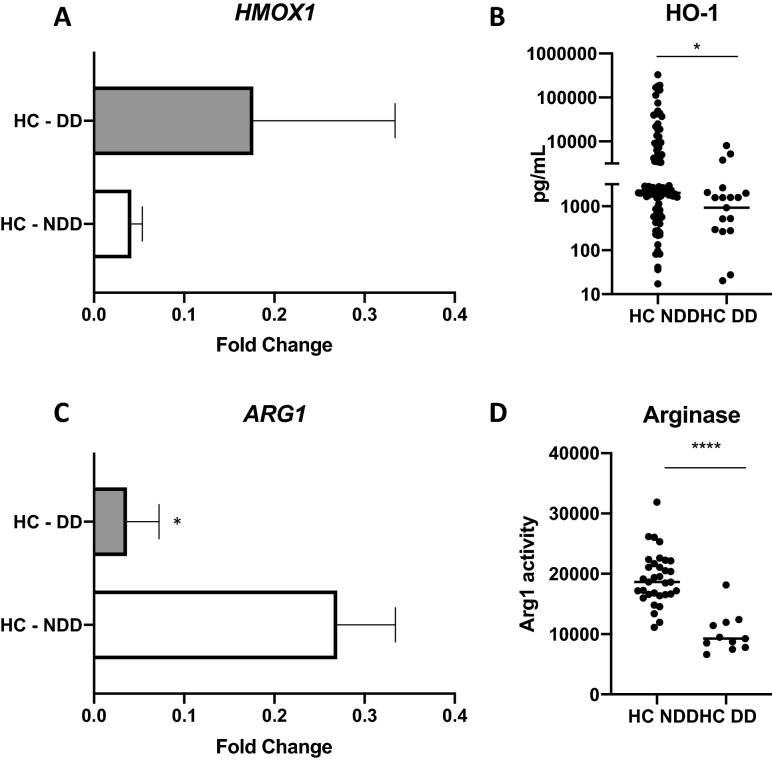
Arginase activity is associated with protection against leprosy in contacts. Whole blood cells and serum from 141 contacts were evaluated. Of these contacts, 33 developed leprosy during the follow-up (HC-DD). HC-NDD, household contacts that did not develop disease. (**A**) *HMOX1* expression, (**B**) HO-1 levels in sera, (**C**) *ARG1* expression and, (**D**) arginase activity. *p ≤ 0.05, ****p ≤ 0.0001.

**Figure 3:**
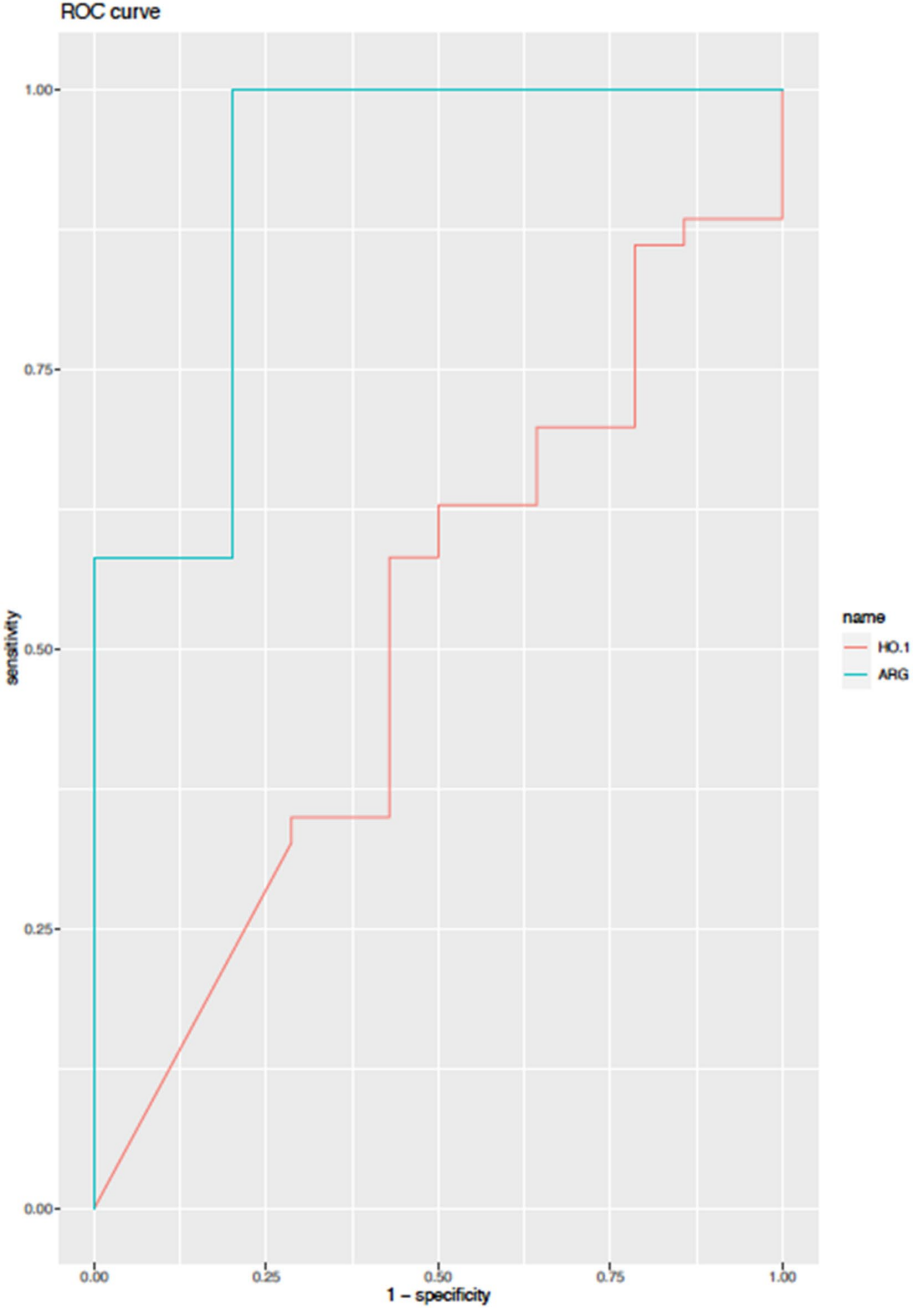
Evaluation of HO-1 and Arginase as predictive markers of illness in leprosy contacts. Receiver operating characteristic curves (ROC) for contacts showing the two markers tested (HO-1, red and Arginase, blue).

A cross-sectional analysis was performed to compare both *ARG1* and *HO-1* expression in samples collected from contacts that developed leprosy (DD) in comparison with contacts that did not develop the disease (NDD). All samples were collected during the first visit at the Outpatient Unit, before the appearance of the first symptoms of the disease. Gene expression of heme oxygenase 1 (*HMOX*) did not significantly differ in samples from contacts that developed the disease (HC-DD) and contacts that did not develop the disease (HC-NDD) (Fig. 2A). In contrast, serum levels of HO-1 were significantly reduced (*p* < 0.05) in the HC-DD group (Fig. 2B). HO-1 was evaluated as a biomarker of pre-clinical leprosy and we observed a 59.26% specificity and a 73.9% sensitivity in distinguishing the HC-DD versus HC-NDD groups (Table 1, Fig. 3). *ARG1* expression was reduced in the whole blood from HC-DD (Fig. 2C) together with a significant reduction in arginase activity in the sera, when compared with the HC-NDD group (*p* < 0.001) (Fig. 2D). Arginase activity presented 90% sensitivity and 96.7% specificity to discriminate HC-DD from HC-NDD (Table 2, Fig. 3).

**Table 1:** Specificity, sensitivity and, accuracy of the use of ELISA of HO-1 to identify contacts more prone to develop leprosy.

ROC Curve (× NDD/DD)				
Feature	Specificity	Sensitivity	Accuracy	AUC
HO-1	0.5925926	0.7391304	0.6183206	0.653
HO-1 vs PGL-1	0.57407407	0.78260870	0.61068702	0.6538
HO-1 vs LID1	0.76851852	0.75572519	0.75572519	0.7496
Ho-1 vs PGL-1 vs LID1	0.58333333	0.86956522	0.63358779	0.754

HO-1 = heme oxygenase 1, PGL-1 = Phenolic glycolipid, LID-1 (the fusion protein product of the ml0405 and ml2331 genes).

**Table 2:** Specificity, sensitivity and, accuracy of the use of arginase activity to identify contacts more prone to develop leprosy.

ROC Curve (× NDD/DD)				
Feature	Specificity	Sensitivity	Accuracy	AUC
ARG1	0.96774194	0.90000000	0.95121951	0.9516
ARG1 vs PGL-1	0.93548387	1.00000000	0.95121951	0.9871
ARG1 vs LID1	0.93548387	1.00000000	0.95121951	0.9871
ARG1 vs PGL-1 vs LID1	0.96774194	1.00000000	0.97560976	0.9903

ARG1 = arginase 1, PGL-1 = Phenolic glycolipid, LID-1 (the fusion protein product of the ml0405 and ml2331 genes).

### Arginase activity combined with serology against PGL-1 and LID-1 increased the sensitivity for discriminating HC-DD from HC-NDD

Since antibodies against *M. leprae* PGL-1 and LID-1 are associated with *M. leprae* exposure, we evaluated if analysis of HO-1 levels or arginase activity combined with *M. leprae*-specific anti-PGL-I or anti-LID-1 antibodies could increase sensitivity in distinguishing HC-DD from HC-NDD. As observed, the combination of HO-1 levels with both positive anti-PGL-1 and anti-LID-1 serological tests increased the sensitivity from 73.9 to 86.9%. Specificity was not affected (Table 1). In the same way, the combination of arginase activity with positive serology for both anti-PGL-1 and anti-LID-1 did not affect specificity, but increased the sensitivity to 100% (Table 2).

## Discussion

Previous studies have demonstrated that the frequent exposure of leprosy contacts to *M. leprae* results in an active innate immune response in these individuals and that the identification of appropriate biomarker signatures can contribute to the identification those at risk of developing leprosy upon *M. leprae* exposure^16,17^.

PB patients and leprosy contacts present similar immune responses. Hooij and colleagues^17^ have suggested that PB leprosy might be a result of an imbalance of the innate immune response in contacts that favor the bacilli. Here, we selected three molecules of skin macrophages that have been associated with the higher susceptibility in MB patients: CD163, HO-1, and arginase 1, and evaluated if there is an association between the disease and an increased systemic anti-inflammatory phenotype in contacts.

Our previous data demonstrated that skin macrophages from MB patients presented increased expression of the scavenger receptor CD163, which can recognize hemoglobin-haptoglobin complexes^14^ and leads to intracellular heme that is the substrate for the enzyme HO-1. This then leads to the production of the anti-inflammatory mediator, CO (carbon monoxide), biliverdin, and increased intracellular iron levels, which could be associated with higher bacillary loads in leprosy patients^15^. In the present study we demonstrated that serum levels of HO-1 were reduced in leprosy contacts when compared with samples from healthy donors. Further to this, the analysis of HO-1 levels in serum from contacts that developed disease during the follow-up were found to be reduced when compared to those from contacts that did not develop disease during the follow-up. These data suggest that in contacts that will develop the disease the bacilli exposure perhaps contributes to a reduction in anti-inflammatory markers systemically, with a more localized immune response.

Arginase 1 is a binuclear manganese metalloenzyme that catalyzes the hydrolysis of arginine to ornithine and urea. Our previous study demonstrated that there is increased expression of arginase in skin cells from MB patients when compared with the PB ones. In addition, we demonstrated that the removal of apoptotic cells by pro-inflammatory macrophages increases arginase expression^13^. Here, we evaluated arginase activity in serum from leprosy contacts and compared that with samples from patients and healthy donors, and it was found to be increased, regardless of whether the index case was MB or PB. This data corroborates the hypothesis that the frequent exposure to *M. leprae* antigens differentially modulates the innate immune response. In addition, the evaluation of arginase expression and activity demonstrated that there was a reduction in both in the group that developed the disease. These data together demonstrate that arginase may be used as a biomarker of protection in leprosy contacts, with 90% sensitivity and 96.7% specificity.

Several reports have evaluated specific antibodies as immune biomarkers of infection. The majority of studies evaluated the specific IgM immune response against PGL-1, a unique cell wall antigen of *M. leprae.* In addition, IgM and IgG antibody responses directed against *M. leprae*-specific recombinant proteins have been evaluated^6,18–20^. Here, we verified that the combined evaluation of HO-1 with positive serology for both PGL-1 and LID-1 increased the sensitivity, but not the specificity, when compared with HO-1 alone, in the capacity to discriminate HC-DD from HC-NDD. The analysis of arginase 1 with the serology increased the sensitivity to 100% while the specificity remained at 96.7% (AUC = 0.99).

Evidence links persistent exposure to *M. leprae* and/or bacillary load in leprosy patients with hyporesponsiveness to *M. leprae*-specific antigens. Although this hyporesponsiveness has been associated with reduced lymphoproliferation, our data suggest that innate pathways might be modulated by *M. leprae* in order to suppress the immune responses that could favor the bacteria instead of the host cells. Previous studies have associated the L-arginine-dependent macrophage effector functions with the metabolic activity of *M. leprae*^21^, but more studies are needed in order to understand the exact meaning of the increase in arginase activity in cells from contacts that did not develop disease. In addition, despite the fact that leprosy contacts constitute a group at a higher risk of developing leprosy, it is well known that only a small percentage will progress to active disease. In the present study we evaluated samples from 33 contacts that developed disease, but validation in larger cohorts would be desirable.

Thus, to sum up, the present data suggest that arginase activity is a marker associated with protection against the disease in contacts from leprosy patients.

## Materials and methods

### Contact and patient samples

All samples were obtained from patients and household contacts attended at the Souza Araujo Outpatient Unit (Leprosy Laboratory, Fiocruz, Rio de Janeiro, Brazil). Serum samples from healthy donors from an endemic area (n = 18, age range = 37–63, male/female (%) = 37/63), paucibacillary patients (PB, n = 42, age range = 19–71, male/female (%) = 40/60), and multibacillary patients (MB, n = 59, age range = 21–74, male/female (%) = 71/29, mean bacillary index (BI) = 3.25) were evaluated. All patients were recruited at diagnosis, prior to treatment, and they did not exhibit any signs of leprosy reactions.

The samples were obtained and evaluated using protocols approved by the Oswaldo Cruz Institute Research Ethics Committee, with informed consent in writing and signed (CAAE number: 34239814.7.0000.5248). In addition, this research was conducted using approved ethical protocols that were in accordance with the Declaration of Helsinki. Samples from 141 contacts of leprosy patients were evaluated. The individuals of the contact group were further classified into two groups: the household contacts of paucibacillary patients (HC-PB) and the household contacts of multibacillary patients (HC-MB). Thirty-three contacts were diagnosed with leprosy during the follow-up with a mean of 30.24 (2 ± 192) months before illness. The serum levels of HO-1 and the arginase activity were evaluated on the first day of the clinical appointment before the treatment of the leprosy patient, and all the contacts at this time had no clinical indications of leprosy. The contacts that presented previous illness due to cancer, tuberculosis, or any other type of infectious-contagious disease were removed from the study, as well as pregnant women, lactating women, puerperal women, and underage contacts. All characteristics of contacts were included in Supplementary Table 1.

### PAXgene whole-blood RNA extraction and quantitative real-time polymerase chain reaction (qRT-PCR)

Whole blood samples were obtained through venous puncture using PAX gene tubes (Qiagen, Hilden, Germany) from leprosy patients and household contacts. Total RNA was isolated using the PAXgene™ Blood RNA Kit (Qiagen), handled according to the manufacturer’s instructions. The total RNA concentration was quantified on a Nanodrop ND-1000 spectrophotometer (NanoDrop, Wilmington, DE, USA). Standard denaturing agarose gel electrophoresis was performed to determine RNA integrity visualized on a UV transilluminator. cDNA synthesis was carried out using the Superscript III RT-PCR Kit (Applied Biosystems, Branchburg, NJ, USA). RT-qPCR was performed using a final volume of 10 μL containing 10 ng of cDNA, TaqMan Fast Universal PCR Master Mix (2x) (ThermoFisher Scientific, Waltham, MA, USA), and 1X of each TaqMan designed probes. All assays were performed in duplicate for each amplification reaction and a 'no reverse transcriptase control' and 'no template control' were incorporated into each run. Briefly, PCR was performed in a StepOnePlus Real-Time PCR System (Applied Biosystems, Waltham, MA, USA) at 95 °C for 20 s, 40 cycles of 95 °C for 1 s, and 60 °C for 20 s. The studied genes were heme oxygenase 1 (*HMOX1*; Hs01110250_m1) and arginase (*ARG1,* Hs00163660_m1). Glyceraldehyde-3-phosphate dehydrogenase (GAPDH; Hs99999905_m1) was adopted as a reference gene and mRNA was quantified using the 2^−ΔCt^ method.

### ELISA for HO-1 and soluble CD163 serum concentration

Serum was collected from contacts and patients evaluated in the study and samples were stored at −20 °C until use. The concentrations of HO-1 and soluble CD163 (sCD163) were evaluated by the Human Total HO-1/HMOX1 DuoSet IC ELISA Kit (R&D Systems, Minneapolis, MN, USA) and the Human CD163 DuoSet ELISA Kit (R&D Systems) respectively, according to the manufacturer's instructions.

### Arginase activity

The arginase activity was determined by measuring the conversion of L-arginine to L-ornithine and urea using the micro-method described elsewhere^22^. Briefly, 25 μL of serum sample was solubilized with 25 μL of lysis buffer containing 0.1% Triton X-100, 10 mM MnCl_2_, and 50 mM Tris–HCl (pH 7.5). Arginase was activated by heating for 7 min at 56 °C. L-arginine hydrolysis was done by incubating the activated lysates with 50 μL of L-arginine (pH 9.7) at 37 °C for 60 min. The reaction was stopped by the addition of 400 μL acid solution [H_2_SO_4_ (96%)/H_3_PO_4_ (85%)/H_2_O, 1:3:7, v/v/v]. Urea concentration was measured at 540 nm after addition of 20 μL of α-isonitrosopropiophenone (ISPF, dissolved in 100% ethanol; Sigma, St. Louis, MO, USA) using a spectrophotometer (TECAN, USA) followed by heating at 100 °C for 45 min. One unit of enzyme (ARG) activity is defined as the amount of enzyme that catalyzed the formation of one μMol of urea per 60 s.

### Anti-PGL-1 and anti-LID-1 quantification by ELISA

The 96-well plates (Corning, New York, NY, USA) were coated with saturating amounts of either ND-O–BSA (BEI Resources) (0.25 µg/mL) or recombinant LID-1 protein (the fusion protein product of the ml0405 and ml2331 genes, at 1 µg/mL; donated by Dr. Malcolm Duthie—University of Washington) in 0.05 M Na_2_CO_3_/NaHCO_3_ buffered solution prepared in phosphate buffered saline (PBS) overnight at 4 °C. Wells were then washed with PBS/Tween-20 (0.3%). For anti-PGL-1 quantification, wells were blocked with 3% bovine serum albumin (BSA)/PBS/Tween-20 for 1 h at 37 °C, then serum samples diluted 1:200 in 1% BSA/PBS/Tween-20 were added and the plate was incubated for 1 h at 37 °C. For anti-LID-1 quantification, wells were blocked with 1% BSA/PBS/Tween-20 for 1 h at room temperature, then the serum samples diluted 1:200 in 0.1% BSA/PBS/Tween-20 were added and incubated for 2 h. Samples were tested in duplicate. Wells were washed and incubated with anti-human IgM (HRP) peroxidase antibody (1:10,000; Sigma) diluted in 1% BSA/PBS/Tween-20 for anti-PGL-1 quantification or with anti-human IgG (HRP) peroxidase antibody (1:30,000; Rockland Immunochemicals, Gilbertsville, PA, USA) diluted in 0.1% BSA/PBS/Tween-20 for anti-LID-1 quantification for 1 h at room temperature. After washing, plates were incubated with 3,3′,5,5′-tetramethylbenzidine and hydrogen peroxide in a citric acid-citrate buffer (peroxidase color substrate) and for the reaction stop, 1 N H_2_SO_4_ was used. The optical density (OD) of each well was read at 450 nm using a Spectra Max 190 microplate reader (Molecular Devices, Sunnyvale, CA, USA). Results from each individual serum were expressed as the mean OD of their duplicate wells. To be considered valid, the average OD of the positive controls had to be between 0.7–1.3 and the average OD of the negative controls below 0.15. An OD > 0.3 was defined as a positive response, as described by Duthie et al.^23^.

### Analysis plan

The experimental data obtained and the data collected from the medical records were organized and structured into spreadsheets in Microsoft Excel. The analysis between the tests (sensitivity, specificity, accuracy, area under the curve, and p-value) was performed using software R (RStudio, version 3.6.0). Contingency tables were used to compare the results obtained from the study.

### Statistical analysis

All results are shown as the median or mean ± standard error. First, the Kolmogorov–Smirnov test was performed to observe if the samples had a normal distribution, after which the significant differences between the groups were determined through the t-test or the Mann–Whitney test. The statistical analyses and graphs were performed using GraphPad Prism version 8.0 (GraphPad Software). Parallel to this, using the RStudio program, the data were evaluated under a baseline characteristic (progression for the disease) against the hypothesis test variables, where the cross-tabulation of the variables was obtained, resulting in the distribution of the variables and statistical computation of the different subgroups. After this, a logistic regression was used to model and estimate the relationship between the dependent and independent variables, thus calculating the probability of an outcome (DD) belonging to a particular variable. After this definition, an ROC curve was constructed to evaluate the specificity, sensitivity, accuracy, area under the curve, odds ratio, and p-value of the cross variables.

## Supplementary Information

Supplementary Information.
